# Learning to manage tracheostomy-related emergencies: a pilot study comparing three teaching strategies for junior doctors in intensive care

**DOI:** 10.1186/s12909-026-09056-3

**Published:** 2026-03-25

**Authors:** Marie Guinat, Olivier Pantet, Cécile Salathé, Géraldine Stieger, Dan Carel, David Gachoud, Lise Piquilloud

**Affiliations:** 1 Adult Intensive Care Unit, University Hospital and University of Lausanne (CHUV), Rue du Bugnon 46, Lausanne, 1005 Switzerland; 2 Medical Education Unit of the School of Medicine, Faculty of Biology and Medicine, University of Lausanne, Lausanne, Switzerland; 3 Adult Intensive Care Unit, Neuchâtel Hospital, Neuchâtel, Switzerland; 4 Medical Intensive Care, University Hospital Mannheim, Mannheim, Germany; 5 Department of Internal Medicine, Lausanne University Hospital (CHUV), Lausanne, Switzerland

**Keywords:** Tracheostomy, Low-frequency and high-stakes critical event, Teaching strategies, E-learning, Simulation, Flipped classroom, Medical education

## Abstract

**Background:**

In intensive care settings, doctors face low-frequency and high-stakes events such as tracheostomy-related emergencies. Junior doctors must learn to manage these situations to ensure patient safety, yet they often lack clinical exposure to such critical events. Clinical teachers should develop methods that provide enough learning opportunities while remaining feasible in terms of workload. This study compared three strategies for teaching junior doctors to manage tracheostomy-related emergencies in intensive care unit (ICU).

**Method:**

In this pilot study, conducted in the ICU of Lausanne University Hospital, twenty-four junior doctors were randomized into three groups: Group A: Access to a printed algorithm; Group B: E-learning, including the algorithm with access to a toolbox; Group C: Flipped classroom course, including a short low-fidelity simulation. Performance was assessed through pre- and post-intervention sessions consisting of three tracheostomy-related simulation scenarios. A modified Delphi method was used to develop a performance assessment tool consisting of an evaluation scale for each scenario. Knowledge was also assessed before and after the intervention through a multiple-choice questionnaire. The primary outcome was the Global Performance Score (GPS), calculated for each teaching method by summing the performance scale scores across the three simulation scenarios as the total score across all scenarios, after the intervention.

**Results:**

The GPS significantly improved after the intervention in the three groups (Group A: *M* = 141, *SD* = 22 vs. *M* = 116, *SD* = 16.7 *p* = 0.008; Group B: *M* = 157, *SD* = 14 vs. *M* = 110.1, *SD* = 17.3 *p* = 0.008; Group C: *M* = 171.9, *SD* = 20.1 vs. *M* = 99.4, *SD* = 16 *p* = 0.008). There was a trend for a higher post-intervention GPS in Group B as compared to Group A (*p* = 0.105), and in Group C as compared to Group B (*p* = 0.279). By contrast, post-intervention GPS was significantly higher in Group C as compared to Group A (*p* = 0.021).

**Conclusion:**

The flipped classroom strategy, combined with a brief simulation, yielded the highest post-intervention score. This strategy appears promising for junior doctors learning to manage low-frequency critical events, such as tracheostomy-related emergencies. Further research should explore its feasibility at larger scale, confirm the results in a larger population of learners with adequately powered studies and the applicability of such a teaching approach to other critical events.

**Supplementary Information:**

The online version contains supplementary material available at 10.1186/s12909-026-09056-3.

## Background

Intensive Care Unit (ICU) departments are areas of clinical practice where doctors face low-frequency and high-stakes critical events, such as tracheostomy-related adverse events. The low frequency of these potentially life-threatening events explains why doctors in training have limited exposure to them, highlighting the need for effective, practical teaching strategies for these critical events. Regarding tracheostomy, it is a procedure commonly performed in 5 to 25% of mechanically ventilated adult ICU patients [1–3]. While complications related to airway management in tracheostomized critically ill patients are infrequent, they can result in brain injuries and have a high mortality rate, up to 60% [4]. Because these complications may also occur during night shifts, when medical staff may be less experienced [5] and have knowledge gaps in managing such cases [6], recommendations for managing tracheostomy-related emergencies have been developed [7–9]. However, education is pivotal for teaching and training the skills needed to implement these recommendations [7].

Structured teaching outside the clinical environment is essential for doctors in training to acquire the skills to manage the low-frequency, high-stakes critical events [10–12]. The optimal method of teaching this content has not yet been identified and must account for the fact that medical skills training is challenged by constraints such as duty-hour restrictions, shift work, and night-float rotations [13, 14]. To address these challenges, a common approach in emergency clinical contexts is to implement cognitive aids, such as algorithms or pocket cards. It is a low-cost strategy that reaches a large audience but seems insufficient to teach complex care situations [15]. In addition, there is strong evidence that curricula should offer more hands-on experiences related to acute care management [16, 17]. Simulation-based education provides opportunities to expose learners to realistic acute care situations [18], such as high-risk, low-volume critical events [19, 20], without compromising patient security and safety [21]. Nevertheless, these simulated hands-on experiences must be systematically designed to facilitate transfer and be underpinned by educational theory to support optimal competencies development [16, 22]. A whole simulation program to teach every topic relevant to critical care is time-consuming, with high cost and logistical requirements [20]. In that sense, innovative educational approaches should be developed to align with learners’ needs, the exponential growth in medical intensive care skills, time constraints in the ICU environment, and variability in clinical exposure [23]. Technology-based teaching has become an alternative to the traditional apprenticeship model and can help address some of the educational challenges described previously [24]. Online learning offers flexibility in delivering medical training [25], with a growing interest in emergency teaching programs [26, 27]. While online teaching approaches can effectively achieve learning outcomes independently, they are often integrated into blended teaching approaches that combine e-learning technologies with traditional instructor-led training, including simulation [28]. The flipped classroom is an innovative blended teaching approach that provides learners with online didactic content before using face-to-face teaching to apply their knowledge through active teaching and practice [29]. Most publications on flipped classrooms focus on undergraduate education. Recently, authors have highlighted that this approach may be promising for teaching emergency care management in a postgraduate context, where critical care knowledge is required before engaging in more complex clinical problem-solving and practice [30–32]. The positive impact of this learning approach comes with considerable human resources, high costs, and the need for protected teaching time [33], specifically if the simulation is used for in-class activities [34]. Conversely, online learning is described as an economical option to deliver educational content [35]. By analogy, even if studies on managing high-risk, low-volume critical events are rare, the intensive care environment and the acquisition of the specific skills needed to manage emergency situations in ICU patients might be a target for such teaching strategies. Our hypothesis is that the flipped classroom and e-learning are more efficient than providing residents with only cognitive aids to manage low-volume and high-risk critical events related to tracheostomy. The study aimed to compare three approaches to teaching residents how to manage tracheostomy-related emergencies in the ICU.

## Method

### Design and research setting

This prospective pilot study, comparing three teaching methods, was conducted in the Intensive Care Unit (ICU) of the Lausanne University Hospital (CHUV), Switzerland. The ICU at CHUV is a 35-bed multidisciplinary ICU department within a tertiary healthcare centre in Switzerland. This study was conducted in accordance with the ethical principles outlined in the Declaration of Helsinki. All participants provided their written informed consent. This study was approved as exempt by the local Ethics Research Committee, the Cantonal Commission for Ethics in Human Research of the Canton of Vaud, Switzerland (CER-VD, 12 April, 2021), as no patient was included and no medical procedure tested. All procedures were performed in accordance with the ethical standards of the institution, University Hospital of Lausanne (CHUV), Lausanne, Switzerland.

### Participants

Participants were recruited from a group of graduate doctors beginning their residency program and on a six-month ICU rotation at the time of the study. The study was presented on the first day of their ICU clerkship, and all potential participants then received a written invitation to participate, including a detailed explanation of the research’s purpose and the three educational approaches that had to be compared. They enrolled on a voluntary basis. Participants were randomly assigned to one of the three teaching groups based on a computer-generated randomization list. Allocation concealment was ensured through sequentially numbered, opaque, sealed envelopes (SNOSE) prepared in advance by an independent researcher. After written informed consent and completion of baseline assessments for knowledge and performance, the enrolling researcher (MG), responsible for implementing the teaching methods, manually opened the envelope in numerical order to reveal the assignment. Given the small sample size and participants’ heterogeneity, stratification based, for example, on the previous level of experience or knowledge, was not performed. Blinding of the participants was not feasible because they were aware of the teaching they received.

### Intervention

The duration of the teaching intervention was six months. Two doctors (MG and OP) and one nurse (DC) from the ICU department, with expertise in simulation, intensive care, airway management, and medical education, designed the online and flipped classroom courses. The three group interventions were:

- Group A: control group: participants received a pocket card describing the algorithm called “Algorithm to manage respiratory distress in a patient with a tracheostomy”. The pocket card was also available online and at each patient’s bedside in the ICU department of the CHUV. This algorithm was designed by ICU airway management experts based on published guidelines and clinical experience.
- Group B: E-learning with open access to a toolbox: participants attended a two-hour e-learning module designed in accordance with web-based learning principles [36, 37]. This e-learning included the algorithm called “algorithm to manage respiratory distress in a patient with a tracheostomy”, an online lecture consisting of a narrative slide presentation and access to practical guidelines, and a video. In addition to e-learning, participants had access to a toolbox containing all the equipment needed for practical exercises. Participants were provided with three paper-based scenarios to reflect on how to manage clinical situations. The learners received the teaching material program by email at the beginning of the course, and no online platform was used. The estimated total training duration was two hours and thirty minutes.
- Group C: Flipped classroom course including a short low-fidelity simulation course: participants were trained for a total of two hours of teaching consisting of pre-class activities (the same e-learning previously described, including the algorithm called “algorithm to manage respiratory distress in a patient with a tracheostomy”, an online lecture consisting of narrative slide presentation and access to practical guidelines, and a video) followed by in-class activities (low-fidelity simulation course) based on flipped classroom principles [38, 39]. The first author (MG) delivered the low-fidelity simulation course, consisting of one-to-one mentored training on a low-fidelity manikin for 30 to 45 min. This simulation training session followed the four steps of Kolb’s experiential cycle [40]. The estimated total training duration was two hours and thirty minutes.

### Outcomes and evaluation

The main outcome was the Global Performance Score (GPS) for managing tracheostomy-related adverse events in the ICU, calculated as the total score across all practical simulation scenarios (detailed evaluation process described thereafter). The secondary outcomes were the Performance Score for each scenario, the improvement in GPS between the pre-intervention and the post-intervention for each teaching approach, the Knowledge Score, and the improvement in Knowledge Score between the pre-intervention and the post-intervention for each teaching approach.

**Fig. 1:**
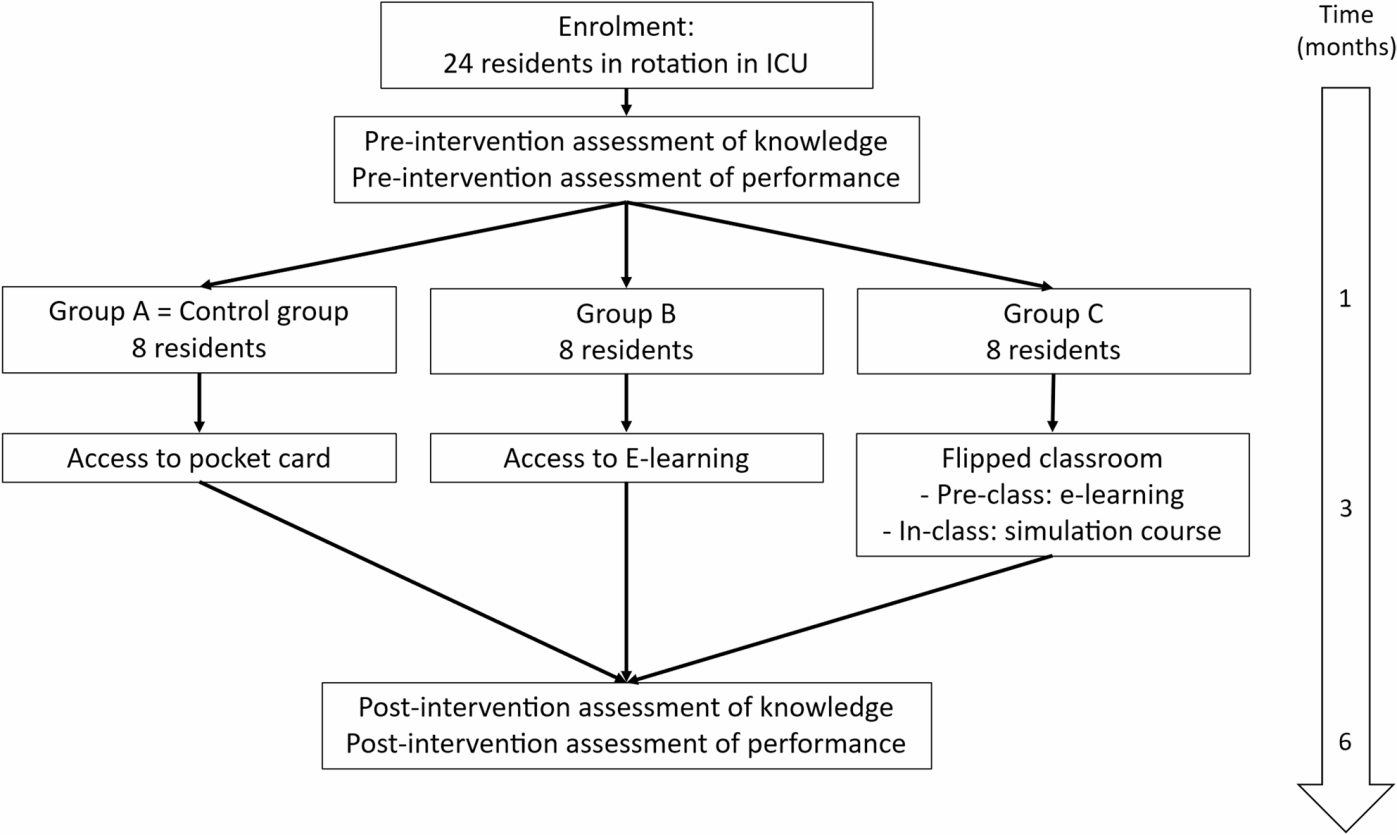
Flow diagram of participants who underwent the study with knowledge and performance assessment before and after the training

Each participant was assessed on performance and knowledge at the beginning and at the end of the teaching intervention period, corresponding to the first and sixth months of ICU rotation (Fig. 1).

### Performance assessment - simulation-based summative assessment scale (SBSA scale)

For the performance assessment, all participants were exposed to the same three low-fidelity simulation scenarios before and after the teaching program according to the randomization group.

#### Simulation scenario development

An educational group composed of two physicians and one nurse (MG, OP, DC) in charge of simulation training in the ICU developed three scenarios to assess residents’ performance in managing tracheostomy-related emergencies in the ICU setting. Scenarios were developed for the study following the principles of constructive alignment [41], key principles of SBSA [42], and based on a literature review and international recommendations on the management of tracheostomy-related events [7, 9].

#### Performance assessment scale development

We did not find an appropriate performance assessment scale in the literature, so we developed a dedicated assessment scale for the study. Based on the three evaluation scenarios, the assessment scale was developed following five steps using a modified Delphi method for content validity [43]. Supplementary file 1 outlines the steps of the modified Delphi method used to develop the three assessment scales (one for each simulation scenario). A pilot testing phase was conducted prior to the final use of the assessment scales to assess the clarity of the scales, the observability of each item, and the appropriateness of the binary scoring (“performed” or “not performed”). Inter-rater reliability was subsequently evaluated by two assessors for five learners. For each scenario, the assessment scale enumerates the key steps the learner needs to take to manage the patient, including, for example, situation assessment, decision-making, and technical task execution. Representative examples of items include “He/She prepares for possible hemodynamic deterioration” or “He/She administers oxygen appropriately (via the patient’s face and tracheostomy)”. The final English version of the performance assessment scales for the three simulation scenarios is provided in Supplementary file 2.

#### Assessment of skills with the performance assessment scale

Before being assessed through each scenario, before and after the teaching period, each participant received the same briefing on the low-fidelity manikin. The briefing included an explanation of the simulation environment, a presentation of the manikin, and materials to manage the scenario. Each scenario began with a resident “being called to the ICU” to manage an acutely ill tracheostomized patient presenting an acute complication. The assessor provided brief, relevant information about the patient to contextualize the scenario for the resident. Participants were asked to “think out loud” by the assessor to allow the reconstruction of the actions and thoughts of the participants. Each scenario was videotaped by MG. No debriefing was provided at the end of a scenario. The videotapes were scored by an independent reviewer (CS) using the performance assessment scale described above, and these scores were used for the statistical analysis. Performance was defined as the completion of the predefined items on the performance assessment scale. The randomization was blinded for CS. GPS was similarly calculated for each teaching method by summing the marks for each item of the performance assessment scale for each scenario and then summing the score obtained for all three scenarios. The videotapes were scored afterward by a second rater (MG) to assess measurement reliability. MG was not blinded to the randomization groups.

### Knowledge assessment - multiple-choice questionnaire (MCQ) tests

For knowledge assessment, participants completed a MCQ for a maximum of fifteen minutes. We did not find an appropriate questionnaire in the literature to assess participants’ knowledge, so we developed a multiple-choice questionnaire for the study. The final English version of the knowledge questionnaire is available in Supplementary File 3. It was designed by an academic teacher in intensive care medicine with expertise in respiratory disease (LP). The first author (MG) collected the MCQ test results before and after the teaching intervention. The MCQ comprised seven questions, each with only one correct answer. Knowledge scores were computed for each participant by summing the points obtained for each question (one correct answer = 1 point).

### Statistics and analysis

No statistical power calculation was conducted before the study due to the convenience sample of participants available at the time of the study. For data analysis, we used nonparametric tests due to the small sample size.

We compared the pre-intervention (pre-test) GPS, the pre-intervention (pre-test) Knowledge Score and the pre-intervention (pre-test) Performance Score for each scenario of Group B with Group A, as well as Group C with Group A, using a Mann-Whitney U test. We compared the post-intervention (post-test) GPS, the post-intervention (post-test) Knowledge Score and the post-intervention (post-test) Performance Score for each scenario of Group B with Group A, as well as Group C with Group A, using a Mann-Whitney U test. To examine potential learning improvement within each teaching strategy, we compared GPS and the Knowledge Score between the pre- and post-intervention using a Wilcoxon signed-rank test. Statistical significance was set at the *α* = 0.05 level. To evaluate the impact of educational interventions, we calculated the effect sizes using Cohen’s d test, with values interpreted as follows: < 0.2 (negligible), 0.2–0.49 (small), 0.5–0.79 (moderate), and > 0.8 (large) [44]. An interclass correlation test was used to determine the interobserver reliability between the two expert evaluators (CS and MG). Data are represented as mean (M) with standard deviation (SD) or median (Md) with interquartile [first, third quartile]. All analyses were performed using R v4.2.3.

## Results

A total of 24 participants were enrolled. Table 1 shows the characteristics of the participants and their Global Performance score and Knowledge Score before the teaching intervention (pre-test). There were no significant differences in the pre-intervention performance score and knowledge score obtained by group B compared to group A, nor by group C compared to group A. Participants had various backgrounds, including surgery (*n* = 1), anaesthesiology (*n* = 4), and internal medicine (*n* = 15). Some of the participants had multiple specializations. All the planned evaluations could be performed.

**Table 1:** Characteristics of the study participants and comparison of Global Performance Score and Knowledge Score at the pre-intervention

	Group A (Group control) Algorithm *n* = 8	Group B E-learning and toolbox *n* = 8	Group C Flipped classroom with simulation *n* = 8
Postgraduate year			
≤ 4	0	1	1
5	2	0	1
6	0	0	0
7	0	3	2
≥ 8	6	4	4
Age			
< 25	0	0	0
25–30	1	1	2
31–35	4	5	5
> 35	3	2	1
Gender			
Female	5	6	7
Male	3	2	1
Training background			
Internal medicine	6	4	5
Pediatric	0	1	1
Emergency	0	2	0
Surgery	0	0	1
Intensive care	5	4	3
Anaesthesiology	2	1	1
Pretraining Global Performance ScoreMean (SD)			
	116 (16.7) -	110 (17.3) *p* = 1	99.4 (16) *p* = 0.322
Pretraining Knowledge Score (MCQ) Mean (SD)			
	6 (1.2) -	6.2 (0.8) *p* = 1	5.4 (1.2) *p* = 0.161

### Global performance score on the three scenarios

#### Comparison of global performance scores between the e-learning (Group B) and Group control (Group A), and between the flipped classroom (Group C) and group control (Group A)

**Fig. 2:**
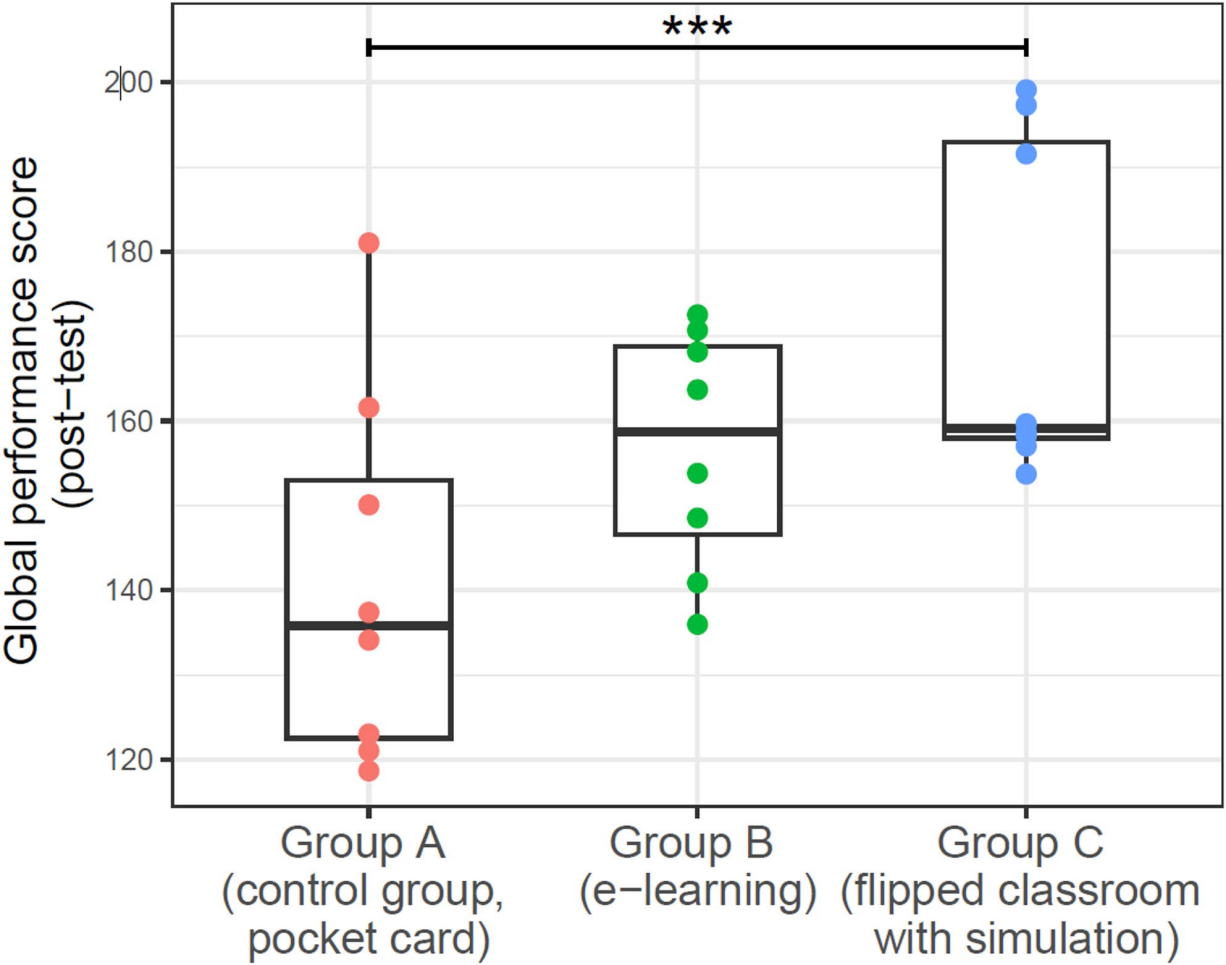
Difference in global performance score between the three educational approaches at the post-intervention. The dots represent the scores obtained by the participants of each group. The horizontal line represents the median, the central box represents the interquartile (25th and 75th percentiles) and the whiskers extend to the smallest and largest score obtained by the participants. The three stars represent statistically significant differences between the scores of the two groups (*p* < 0.05)

Figure 2 illustrates the GPS of participants in the three groups after the intervention. There were no significant differences in the post-intervention performance score obtained by group B (Md = 159 [147–169], M = 157, SD = 14, *p* = 0.105) compared to group A (Md = 136 [123–153], M = 141, SD = 22). However, there was a significant difference in the post-intervention performance score obtained by group C (Md = 159 [158–193], M = 171.9, SD = 20.1, *p* = 0.021) compared to group A (Md = 136 [123–153], M = 141, SD = 22). As a post hoc comparison, we also compared group B and group C, there was no significant difference in the post-intervention performance score obtained by group B (Md = 159 [147–169], M = 157, SD = 14) compared to group C (Md = 159 [158–193], M = 171.9, SD = 20.1, *p* = 0.279).

### Performance score for each scenario

The performance scores of participants in the three groups after the intervention for each scenario are available in the Supplementary file 4.

### Inter-rater reliability of the evaluation

The intra-class correlation coefficient was computed to assess agreement between the two evaluators (CS and MG) in rating the performance of the twenty-four participants. There was a strong agreement between evaluators using the two-way random effect models and “single rater” unit, with a kappa value of 0.977 (95% confidence interval: 0.93–0.99, *p* < 0.05) and 0.952 (95% confidence interval: 0.63–0.99, *p* < 0.05) for the pre- and post-intervention scores, respectively.

### Knowledge score

#### Comparison of knowledge scores between the e-learning (Group B) and group control (Group A), and between the flipped classroom (Group C) and Group control (Group A)

**Fig. 3:**
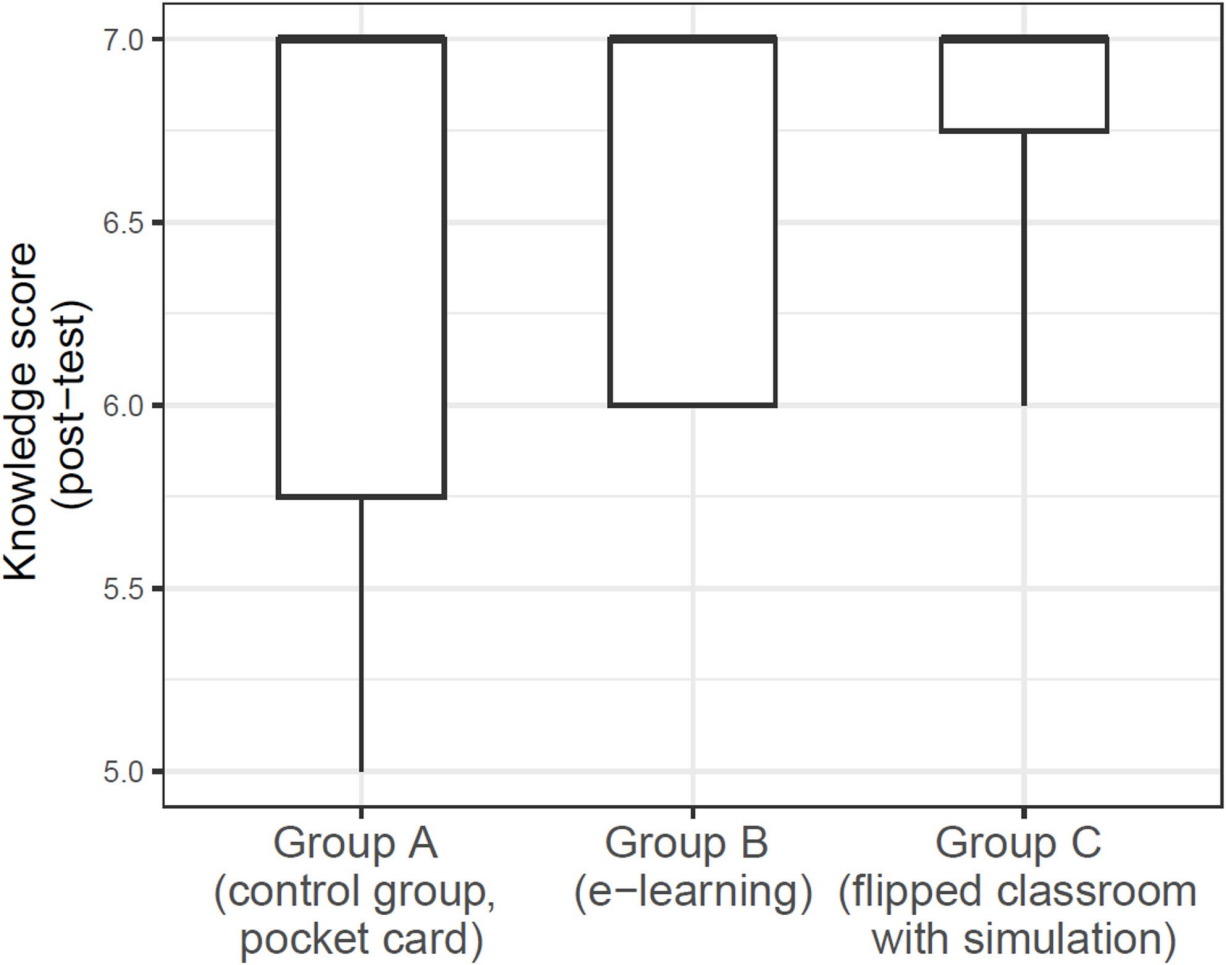
Difference in knowledge score between the three educational approaches at the post-intervention. The dots represent the scores obtained by the participants of each group. The horizontal line represents the median, the central box represents the interquartile (25th and 75th percentiles) and the whiskers extend to the smallest and largest score obtained by the participants

Figure 3 illustrates the knowledge score of participants in the three groups after the intervention.

There was no significant difference in the post-intervention knowledge score obtained by group B (Md = 7 [6–7], M = 6.63, SD = 0.52, *p* = 0.76) compared to group A (Md = 7 [5.75-7], M = 6.38, SD = 0.92). There was no significant difference in the post-intervention knowledge score obtained by group C (Md = 7 [6.75-7], M = 6.75, SD = 0.46, *p* = 0.48) compared to group A (Md = 7 [5.75-7], M = 6.38, SD = 0.92).

### Improvement in global performance score within the three groups

**Fig. 4:**
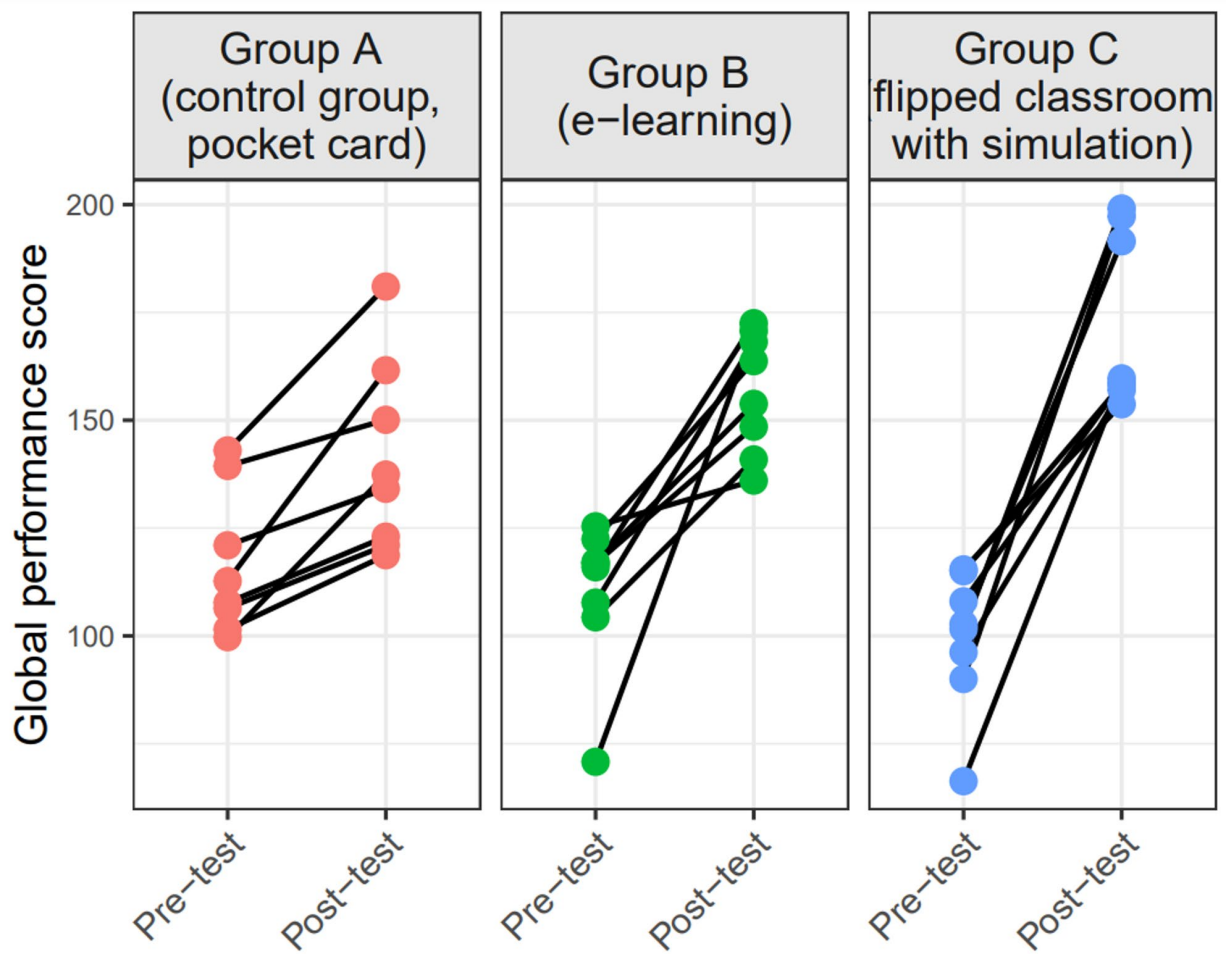
Improvement in global performance score within groups across the three educational approaches. The dots represent the scores obtained by the participants of each group

Figure 4 illustrates the GPS at pre-intervention and post-intervention in each educational group. In the three groups A, B and C, participants’ performance scores on the post-intervention (*M* = 141, *SD* = 22; *M* = 156.8, *SD* = 14; *M* = 171.9, *SD* = 20.1, respectively) were significantly higher than scores on the pre-intervention (*M* = 116, *SD* = 16.7; *M* = 110.1, *SD* = 17.3; *M* = 99.4, *SD* = 16, respectively), showing a small to high effect size (Cohen’s *d* = 0.33; *d* = 1; *d* = 1, respectively).

### Improvement in knowledge scores within the three groups

Table 2 illustrates the change in knowledge score between the pre-intervention (pre-test) and the post-intervention (post-test) for each of the three educational approaches.

**Table 2:** Comparison of knowledge scores between the pre-test and the post-test

Group	Knowledge scores M (SD)		*p*-value	Effect size
	Pre-test	Post-test		
Group A – control group (*n* = 8)	6 (1.2)	6.4 (0.9)	*p* = 0.586	Cohen’s *d* = 0.33
Group B (*n* = 8)	6.1 (0.8)	6.6 (0.5)	*p* = 0.203	Cohen’s *d* = 0.67
Group C (*n* = 8)	5.4 (1.2)	6.8 (0.5)	*p* = 0.034	Cohen’s *d* = 1

## Discussion

The results of this study suggest that the flipped classroom approach with simulation during the in-class period enhances learners’ performance in managing tracheostomy-related adverse events in the ICU when compared with the traditional teaching approach. In our study, the e-learning approach did not lead to improved performance compared with traditional teaching. Regardless of the instructional group, we observed a significant improvement in performance in each group between before and after the intervention. In terms of knowledge, e-learning and flipped classrooms were not more effective compared to traditional learning. We found a significant increase in knowledge between the pre- and post-teaching intervention for the flipped classroom approach only. We observed baseline knowledge score differences between groups, as group C has a lower knowledge score than group A. Even if the difference is not statistically significant, it may affect the magnitude of the observed improvements between pre- and post-intervention. However, by focusing on changes in knowledge scores between pre- and post-test, we attempted to mitigate the potential influence of initial differences between groups.

Our results are consistent with previous studies conducted in the ICU. For example, Yoosoof et al. demonstrated a significant improvement in knowledge and skills acquisition in newborn resuscitation training after participating in a flipped program [45]. In another example in a postgraduate setting, a flipped classroom for teaching point-of-care echocardiography to medical doctors in the surgical ICU increased learners’ knowledge and confidence [34]. Research supports the idea that integrating a short simulation session, rather than a traditional full-day simulation, into a flipped classroom design improves learning [46, 47]. Dong et al. highlighted the importance of pre-class content in facilitating knowledge retrieval during simulations [28]. Considering that the participants in this study are not novices, this flipped classroom was likely built on their prior knowledge to anchor the new information, thereby contributing to better achievement of learning outcomes [48]. In addition, the individual feedback, provided as a “guide to the side” coaching during the short simulation, may encourage problem-solving development and facilitate reflection on the knowledge gap during experiential learning [34, 49].

The results of our study, which did not show a difference in performance gain between e-learning and traditional learning based on algorithm delivery, align with a number of previous studies. Some studies conducted in emergency care settings also demonstrated no statistically significant difference between e-learning and traditional learning for emergency airway management [50], and lung and vascular access ultrasound skills [51, 52]. The high degree of complexity of our learning objectives, which involve managing tracheostomy-related adverse events, may prevent the demonstration of the teaching benefits of e-learning [35]. As a pragmatic approach, we integrated a toolbox into the e-learning, providing concrete, hands-on exposure to materials as a complement to help management of real situations, but we could imagine that the intensity of the intervention was insufficient to show a gain in performance. Another potential explanation for the similar performance outcomes observed between e-learning and traditional teaching could be the lack of participants’ engagement and participation in the online course. Indeed, we did not record the percentage of participants who really took part in the e-learning. In addition, we did not check the time spent doing the e-learning nor how the participants used the educational content. Objective measures of learners’ engagement, such as time spent online, number of logins, course completion rates, or more advanced metrics such as eye-tracking, might help measure adherence [53, 54]. Finally, while learners should be encouraged or supported to engage with e-learning, their adherence to real-life conditions needs careful consideration when implementing education programs in the health professions. Literature highlights that self-regulated learning [55], educational background, students’ motivation, the autonomy of the learners, and spacing-out study of e-learning may influence the learning outcomes [56, 57]. Following the principle of multimedia learning, repeated, self-spaced viewing and iterative review might influence mental model creation and learning gain [58]. In opposition, the flipped classroom approach may motivate participants to engage in online learning before actively engaging in the simulation course [34]. The explanation of the rationale behind the engagement of pre-class assignments facilitates “buy-in” from the learners.

Managing tracheostomy-related adverse events, as a low-volume and high-risk critical event, might be associated with high cognitive load, causing stress and struggles for the learners. The literature indicates that the flipped classroom approach allows better management of cognitive load [59] and increases the intrinsic and extrinsic motivation of the learners [60]. In addition, in-class simulation activities may help learners train high-order skills by integrating knowledge taught during pre-class activities [61, 62]. So, even a brief exposure to experiential learning through simulation courses may reduce cognitive overload related to the learning goals, facilitating reflection and problem-based learning with active exposure to learning opportunities in an almost authentic context [63]. This may help explain the results of this pilot study, which suggest that a flipped classroom approach using simulation-based courses for in-class activities is associated with gains in performance and knowledge. More qualitative research may help to better understand how flipped classroom design might influence further performance in general and cognitive overload in particular.

Finally, regardless of the educational approach, we observed a significant improvement in performance between before and after the intervention in each group. It is interesting to note that clinical exposure during the six months of ICU rotation and access to the algorithm contribute to the improvement of performance in an emergency setting, even if the learning effect is low. This is consistent with some studies that support the use of cognitive aids to improve performance in stressful situations [64, 65]. Nevertheless, across the three teaching approaches, observed performance improvements may reflect not only the effect of the teaching intervention but also learners’ cumulative clinical exposure during a six-month ICU rotation. Repeated exposure to critical events allows learners to consolidate skills in managing unstable patients, thereby enhancing performance independent of the three teaching interventions.

Another explanation of the performance improvement in each group, between the pre-intervention and the post-intervention, is the re-test effect, which refers to the phenomenon in which learners improve their performance not solely due to skills acquisition but also as a result of repeated exposure to assessment scenarios [66]. Post-test performance may partly reflect their memorization of the details of the assessment scenario rather than true mastery of the learning goal. Indeed, repeated exposure to similar scenario assessments increases familiarity with the evaluation process and reduces stress and anxiety. The potential effect of the first exposition to the scenario and the additional time of clinical experience between the two evaluation rounds were however the same for the different groups and cannot thus explain the observed differences in performance recorded. To minimize this bias, the article’s author emphasized the importance of the briefing before engaging the learners in the assessment simulation scenarios, which helps them to better immerse themselves in the assessment environment from the first assessment exposition. This helped ensure that performance during the initial assessment more accurately reflected learners’ baseline performance rather than the novelty effect.

Studies show that many newly graduated doctors perceived themselves as less prepared to manage patients with emergency conditions [67, 68] and present knowledge gaps [69]. Despite this actual shift in the culture of medical education, which promotes more online learning, interactive, collaborative, and learner-centered teaching, the traditional in-class lecture remains one of the most common teaching methods in the intensive care context [70, 71]. Educational designers should prioritize the development of teaching methods that specifically address the educational challenges in the ICU environment and provide safe learning opportunities to care for acutely ill patients [72]. A flipped classroom might represent a solution to modulate the cognitive load associated with clinical experience and the learning process of high cognitive skills.

### Strengths

One strength of the current study is the detailed description of the method used for developing three performance assessment scales using a modified Delphi method [73–76]. In addition, in this study, the flipped classroom approach seemed to be an effective teaching method, as it was associated with a greater increase in GPS.

### Limitations

First, given the novelty of the teaching method and the exploratory nature of this pilot study, a sample size calculation was not performed. The small sample size increases the risk of type 2 error, which is a clear limitation of our work. Second, this pilot study, was limited to a single center and included a small number of participants, which may introduce selection bias and limit the generalisability of the findings. A larger sample is necessary to confirm the results. Third, only a short-term performance assessment was conducted. It would be pertinent to investigate the long-term effect on knowledge retention and performance. Fourth, regarding, the performance assessment scale, although content validity of the assessment scale was done using a modified Delphi method and inter-rater reliability was checked before the study, a more comprehensive evaluation of construct validity should be conducted in future research to definitively validate the evaluation scale. Also, the limited number of MCQ questions reduced the precision of the assessment. Fifth, this study focused only on one specific low-exposure and high-risk event in the ICU; we cannot ensure these results are generalized to another topic. Sixth, regarding other factors that could have influences the results, self-regulated learning may have influenced learners’ motivation to engage in our teaching, particularly for the online course, which could have influence the results. This point was not evaluated in this study. Nevertheless, participating in the study made participants more aware of the subject, which probably positively influenced their learning. Finally, improvement in performance at the post-test may have been partly due to a learning effect arising from the retest effect, given that the same test was taken twice (the same three scenarios). Nevertheless, because no feedback was provided on participants’ performance after the pre-test, we assume this effect was limited. In addition, this potential learning effect from the first exposure was the same across the 3 groups.

### Implications for practice

This study may inform the design of future courses to better address learners’ needs. Using the flipped classroom approach with a short simulation course related to acute care may improve learners’ performance in low-volume and high-risk critical events. This study can guide educators in using these design factors in their course development to address educational challenges, even if further research in larger groups of learners should confirm our results.

### Suggestions for future research

Our current results are promising, but the small sample size of this study highlights the need for larger studies to expand upon these results. In addition, future research should be conducted within the same cohort with a longer follow-up period to evaluate longitudinal retention of these skills after a single intervention. Other studies should evaluate whether the effectiveness of the flipped classroom approach with simulation is confirmed for other intensive care learning outcomes. Moreover, it should be interesting to determine the optimal way to flip the classroom and assess how the specific design of the flipped classroom (e.g., simulation course duration, management of self-regulated learning, modalities of pre-class activities) may influence learning and improve learners’ performance.

## Conclusion

Innovative learning strategies should be designed and implemented to try to achieve learning goals while addressing the educational challenges of the intensive care context. In this pilot study, a flipped classroom approach using simulation for in-class activities improved performance compared to standard teaching, though the small sample size and other potential limitations limits the generalizability of our results. E-learning appears to yield performance gains comparable to those of traditional learning. However, in our study learners’ adherence to e-learning was not assessed, which may have impacted the efficacy of e-learning in improving the tested skills. We encourage the development of a blended approach in intensive care to teach low-volume, high-risk critical events. More research is needed to investigate the impact of instructional design on patient quality of care and on how learners transfer skills to the bedside.

## Supplementary Information

Supplementary Material 1.

Supplementary Material 2.

Supplementary Material 3.

Supplementary Material 4.

## Data Availability

The datasets generated during the current study are available from the corresponding author upon reasonable request.

## Abbreviations

CVI: content validity index
GPS: Global Performance Score
I-CVI: Item content validity index
S-CVI/Ave: Scale Level Content Validity Index/Average
S-CVI: Scale Level Content Validity Index
ICU: intensive care unit
M: Mean
SD: standard deviation
Md: median
