# Supplementary material for: Learning to manage tracheostomy-related emergencies: a pilot study comparing three teaching strategies for junior doctors in intensive care
Source: BMC Med Educ. 2026 Mar 25;26:713. doi: 10.1186/s12909-026-09056-3 (PMC13137732; doi:10.1186/s12909-026-09056-3)
Supplement: Supplementary file 1 — Supplementary Material 1. [file 12909_2026_9056_MOESM1_ESM.docx]

**Supplementary file 2**

Performance assessment scale

**Performance Assessment scale for each scenario**

| **Brief description of scenario 1:**  During a low-fidelity mannequin simulation, the learner takes charge of a patient who has been tracheostomized for a week (in the context of prolonged ventilatory withdrawal following resolved cardiogenic shock). The patient is sedated, curarized, and ventilated under volume control due to pneumonia. The patient desaturates at 80% on 65% Fi02. The tracheostomy cannula is a non-fenestrated cuffed cannula.  Problem and solution: The patient desaturates because sticky secretions obstruct the internal tracheostomy cannula. | **Performed by the learners (yes = 1 point or no = 0 point)** |
| --- | --- |
| **Item of the performance assessment scale** |  |
| 1. He/She recognizes the desaturation |  |
| 2. He/She monitors hemodynamic parameters |  |
| 3. He/She prepares for possible hemodynamic deterioration |  |
| 4. He/She assesses the patient for spontaneous breathing |  |
| 5. He/She calls for help in a manner appropriate to the seriousness of the situation (2nd person/heart alarm) |  |
| 6. He/She requests that the difficult intubation trolley be made available nearby. |  |
| 7. He/She prioritizes restoration of oxygenation with administration of 100% Fi02. |  |
| 8. He/She administers oxygen appropriately (via tracheostomy). |  |
| 9. He/She checks the ventilator circuit connection |  |
| 10. He/She checks ventilator data |  |
| 11. He/She checks the patient's auscultation |  |
| 12. He/She positions the patient's head to facilitate access to the tracheostomy. |  |
| 13. He/She identifies the type of tracheostomy cannula (non-fenestrated with inflated cuff) |  |
| 14. He/She prepares equipment for suctioning (gloves, endotracheal suction catheter, and Yankauer) |  |
| 15. He/She aspirates through the internal cannula with the endotracheal suction probe |  |
| 16. He/She disconnects the ventilator, removes the internal cannula, and sucks into the external cannula with an endotracheal suction catheter |  |
| 17. He/She inserts a new internal cannula adapted to the situation |  |
| 18. He/She ventilates the patient with the bag mask valve on the tracheostomy. |  |
| 19. He/She inspects the capnography curve |  |
| 20. He/She aspirates at the back of the mouth |  |
| 21. He/She checks the balloon pressure |  |
| 22. He/She resumes mechanical ventilation on tracheostomy |  |
| 23. He/She uses equipment appropriately |  |

| **Brief description of scenario 2:**  During a simulation with a low-fidelity mannequin, the learner takes charge of a patient who has been tracheotomized for a week due to a pathological awakening (prolonged ventilatory weaning post-cerebral hemorrhage). The patient is not sedated, with pressure support ventilation, and is currently in ventilatory weaning with pressure at 12, positive end-expiratory pressure at 5, and Fi02 at 30%. The patient desaturates at 80% on 30% Fi02. The tracheostomy cannula is a non-fenestrated cuffed cannula.  Problem and solution: The patient desaturates because the cuff is deflated due to a puncture. | **Performed by the learners (yes = 1 point or no = 0 point)** |
| --- | --- |
| **Item of the performance assessment scale** |  |
| 1. He/She recognizes the desaturation |  |
| 2. He/She monitors hemodynamic parameters |  |
| 3. He/She prepares for possible hemodynamic deterioration |  |
| 4. He/She calls for help in a manner appropriate to the severity of the situation (2nd person) |  |
| 5. He/She assesses whether the patient is breathing spontaneously. |  |
| 6. He/She requests that the difficult intubation cart be made available nearby. |  |
| 7. He/She prioritizes restoration of oxygenation by administering 100% Fi02. |  |
| 8. He/She administers oxygen appropriately (via the patient's face and tracheostomy). |  |
| 9. He/She checks the ventilator circuit connection |  |
| 10. He/She checks ventilator data (exhaled/inspired volume alarms) |  |
| 11. He/She checks the patient's auscultation |  |
| 12. He/She positions the patient's head to facilitate access to the tracheostomy. |  |
| 13. He/She identifies tracheostomy cannula type |  |
| 14. He/She listens for tracheostomy leaks |  |
| 15. He/She prepares suction equipment (gloves, endotracheal suction catheter, and Yankauer). |  |
| 16. He/She aspirates at the back of the mouth |  |
| 17. He/She sucks into the internal cannula with the endotracheal suction probe |  |
| 18. He/She assists the patient with the bag valve mask on tracheostomy |  |
| 19. He/She inspects the capnography curve |  |
| 20. He/She checks cuff pressure |  |
| 21. He/She attempts to reinflate the balloon with a manometer and/or syringe. |  |
| 22. He/She suggests calling ENT for transcanulation |  |
| 23. He/She resumes mechanical ventilation on the tracheostomy while waiting for the ENT. |  |
| 24. He/She maintains oxygenation by face mask and 100% Fi02 on the ventilator. |  |
| 25. He/She explains care to the patient |  |
| 26. He/She observes asepsis |  |
| 27. He uses appropriate equipment |  |

| **Brief description of scenario 3:**  During a simulation with a low-fidelity mannequin, the learner takes charge of a patient who has been tracheostomized for a week (in the context of prolonged ventilatory withdrawal post-ARDS resolution). The patient is conscious, not sedated, and discharged with a 45% Fi02 on tracheostomy, cuff deflated. He has been trans-cannulated for a fenestrated external cannula. The patient is 80% desaturated.  Problem and solution: The patient desaturates because the tracheostomy cannula is in a false channel. | **Performed by the learners (yes = 1 point or no = 0 point)** |
| --- | --- |
| **Item of the performance assessment scale** |  |
| 1. He/She recognizes the desaturation |  |
| 2. He/She monitors hemodynamic parameters |  |
| 3. He/She prepares for possible hemodynamic deterioration (syringes of atropine and phenylephrine available) |  |
| 4. He/She calls for help in a manner appropriate to the seriousness of the situation (2nd person/resuscitation alarm). |  |
| 5. He/She assesses whether the patient is breathing spontaneously. |  |
| 6. He/She requests that the difficult intubation cart be made available nearby. |  |
| 7. He/She prioritizes restoration of oxygenation by administering 100% Fi02. |  |
| 8. He/She administers oxygen appropriately (via face and tracheostomy). |  |
| 9. He/She checks the oxygen connection |  |
| 10. He/She checks the patient's auscultation |  |
| 11. He/She positions the patient's head to facilitate tracheostomy access |  |
| 12. He/She identifies the type of tracheostomy cannula (fenestrated with cuff). |  |
| 13. He/She prepares suction equipment (gloves, endotracheal suction catheter, and Yankaeur). |  |
| 14. He/She aspirates through the internal cannula with an endotracheal suction catheter and identifies the lack of progression of the suction catheter. |  |
| 15. He/She removes the internal cannula and sucks it into the external cannula with an endotracheal suction catheter (lack of progression persists). |  |
| 16. He/She changes the internal cannula |  |
| 17. He/She checks that the cuff is deflated and identifies the absence of air passage through the tracheostomy and the absence of leaks. |  |
| 18. He/She removes the tracheostomy cannula. |  |
| 19. He/She assists the patient with the bag valve mask at face level, closing the tracheostomy orifice with a compress, or assists the patient with the bag valve mask with a pediatric mask via the tracheostomy orifice. |  |
| 20. He/She calls ENT for recanulation |  |
| 21. He/She explains care to the patient |  |
| 22. He/She observes asepsis |  |
| 23. He/She uses appropriate equipment |  |
