# Supplementary material for: Learning to manage tracheostomy-related emergencies: a pilot study comparing three teaching strategies for junior doctors in intensive care
Source: BMC Med Educ. 2026 Mar 25;26:713. doi: 10.1186/s12909-026-09056-3 (PMC13137732; doi:10.1186/s12909-026-09056-3)
Supplement: Supplementary file 2 — Supplementary Material 2. [file 12909_2026_9056_MOESM2_ESM.docx]

**Supplementary file 3**

Knowledge assessment questionnaire

**Knowledge assessment scale using a multiple-choice questionnaire**

**Question 1:** In accidental decannulation, what is the priority action after the call for help?

A. Reposition the tracheostomy tube

B. Oxygenate

C. Ventilate

D. Sedate

**Question 2:** The following cannulas exist except

A. External fenestrated cannula with balloon

B. Internal fenestrated cannula with balloon

C. External non-fenestrated cannula with balloon

D. Internal non-fenestrated cannula

**Question 3:** The following statement is correct. Anatomically, the trachea :

A. Is composed of 16 to 20 closed, circular cartilaginous rings

B. lies between the larynx and the bronchial tubes

C. Composed internally of non-ciliated mucosa

D. is closed in its anterior part by a muscular structure

**Question 4:** In the presence of a tracheostomy, when the internal cannula is not fenestrated, and the cuff is inflated

A. The patient can speak

B. Airway humidification is physiological

C. Air can only pass through the tracheostomy tube

D. The patient cannot cough up secretions.

**Question 5:** With a tracheostomy cannula, the following configurations are possible except :

A. external non-fenestrated cannula, deflated cuff, internal non-fenestrated cannula, speaking stopper

B. Non-fenestrated external cannula, inflated cuff, non-fenestrated internal cannula, speaking cap

C. External fenestrated cannula, internal non-fenestrated cannula, inflated balloon

D. External fenestrated cannula, deflated balloon, internal fenestrated cannula, talking cap

**Question 6:** When ventilating with an intensive care ventilator on a tracheostomy tube :

A. The ventilator is connected to the internal cannula

B. Ventilation is possible when the cuff is deflated

C. Ventilation is not possible if the external cannula is fenestrated

D. Ventilation requires that the balloon is inflated and either the external or internal cannula is not fenestrated.

**Question 7:** Balloon inflation :

A. Prevents micro-aspiration

B. Allows phonation rehabilitation to begin

C. Does not allow ventilation

D. Avoids macro-aspiration
