# Supplementary material for: Learning to manage tracheostomy-related emergencies: a pilot study comparing three teaching strategies for junior doctors in intensive care
Source: BMC Med Educ. 2026 Mar 25;26:713. doi: 10.1186/s12909-026-09056-3 (PMC13137732; doi:10.1186/s12909-026-09056-3)
Supplement: Supplementary file 3 — Supplementary Material 3. [file 12909_2026_9056_MOESM3_ESM.docx]

**Supplementary file 4**

Difference in performance score between the three educational approaches at the post-intervention for each scenario

This figure illustrates the performance scores of participants in the three groups after the intervention for each scenario.


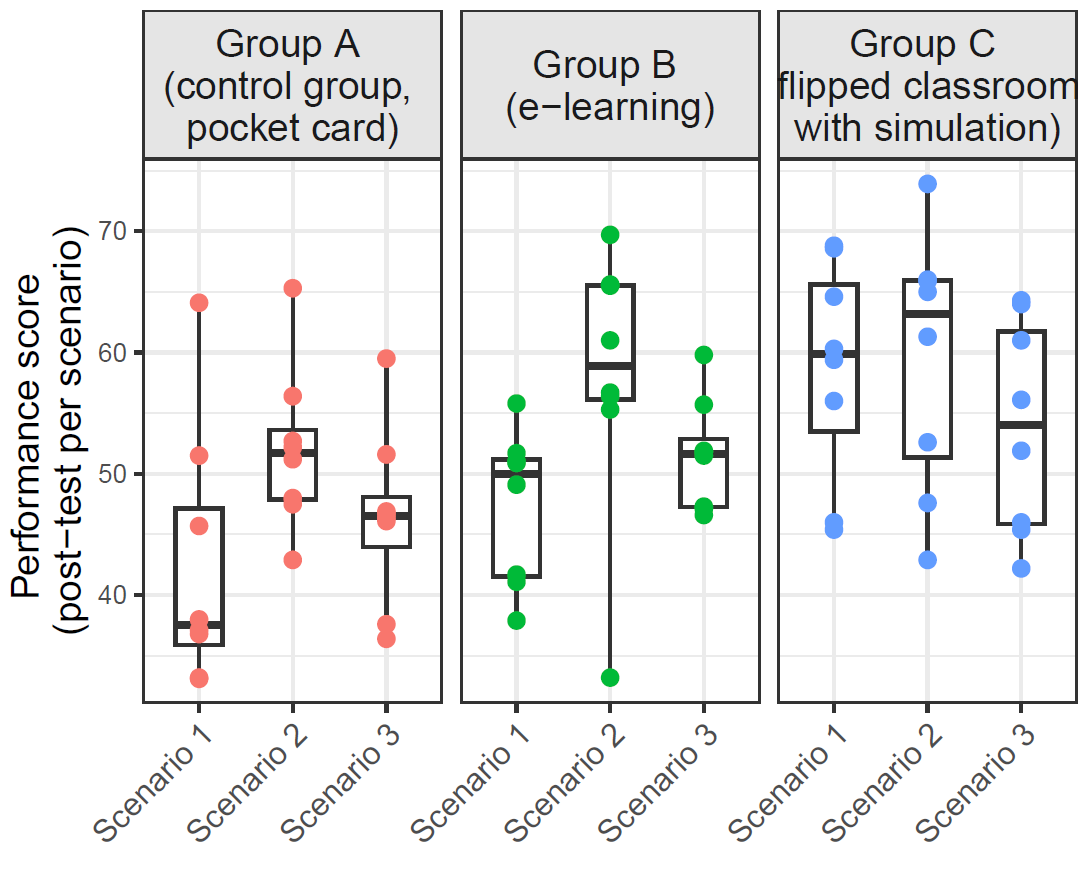


**Additional file 3.** **Difference in performance score between the three educational approaches at the post-intervention for each scenario**. *The dots represent the scores obtained by the participants of each group. The horizontal line represents the median, the central box represents the interquartile (25^th^ and 75^th^ percentiles) and the whiskers extend to the smallest and largest score obtained by the participants.*

There was no significant difference in the pre-intervention performance score attained by group A (group control) and group B (e-learning) (p=0.798) for scenario 1. There was no significant difference in the pre-intervention performance score attained by group A (group control) and group C (simulation) (p=0.103) for scenario 1. There was no significant difference in the pre-intervention performance score attained by group A (group control) and group B (e-learning) (p=0.958) scenario 2. There was no significant difference in the pre-intervention performance score attained by group A (group control) and group C (simulation) (p=0.065) for scenario 2. There was no significant difference in the pre-intervention performance score attained by group A (group control) and group B (e-learning) (p=0.793) for scenario 3. There was no significant difference in the pre-intervention performance score attained by group A (group control) and group C (simulation) (p=0.293) for scenario 3.

There was a significant difference in the pre-intervention performance score obtained by group C (Md = 59.8, M = 5.6, SD = 9.1) compared to group A (Md = 37.5, M = 42.4, SD = 10.8) for scenario 1 (p=0.010).

There was no significant difference in the pre-intervention performance score obtained by group B (Md = 50, M = 47.4, SD = 6.32) compared to group A (Md = 37.5, M = 42.4, SD = 10.8) for scenario 1 (p=0.161).

There was no significant difference in the pre-intervention performance score obtained by group B (Md = 58.9, M = 57.9, SD = 11.3) compared to group A (Md = 51.7, M = 52, SD = 6.7) for scenario 2 (p=0.059). There was no significant difference in the pre-intervention performance score obtained by group C (Md = 63.1, M = 59.4, SD = 10.6) compared to group A (Md = 51.7, M = 52, SD = 6.7) for scenario 2 (p=0.172).

There was no significant difference in the pre-intervention performance score obtained by group B (Md = 51.6, M = 51.5, SD = 4.6) compared to group A (Md = 46.5, M = 46.4, SD = 7.3) for scenario 3 (p=0.05). There was no significant difference in the pre-intervention performance score obtained by group C (Md = 54, M = 53.9, SD = 8.8) compared to group A (Md = 46.5, M = 46.4, SD = 7.3) for scenario 3 (p=0.234).

This indicates that the flipped classroom approach with simulation was more effective than traditional learning regarding performance only for scenario 1. On the contrary, e-learning was not more effective than traditional learning for the three scenarios.
