# Supplementary material for: Learning to manage tracheostomy-related emergencies: a pilot study comparing three teaching strategies for junior doctors in intensive care
Source: BMC Med Educ. 2026 Mar 25;26:713. doi: 10.1186/s12909-026-09056-3 (PMC13137732; doi:10.1186/s12909-026-09056-3)
Supplement: Supplementary file 4 — Supplementary Material 4. [file 12909_2026_9056_MOESM4_ESM.docx]

**Supplementary file 1**

Description of a modified Delphi method to develop three performance assessment scales for three scenarios with low-fidelity simulation

The modified Delphi method is widely used to collect data from experts in their expertise to develop an assessment scale to assess performance [1-4].

# **Step 1: Design of the scale**

Following scenario development, the three researchers of the educational group in the ICU (MG, OP, DC) developed one assessment scale for each scenario. Assessment scales were newly developed for this study, and the full English version of the final assessment scales is available in Supplementary file 2. They indicated different items that the participant must complete to demonstrate successful performance. An iterative process of face-to-face meetings was used to reach a consensus on each item of the assessment scale for each scenario. These scales were tested by the three previous researchers with expertise in simulation training during a simulation course. The final scale for each of the three scenarios was used for the content validity process via the round of the modified Delphi technique as described below [5].

# **Step 2: Judgment and quantification**

Experts panel

We created a heterogeneous group of experts to validate the scale through a modified Delphi method. Experts for the consensus panel were selected based on their background in airway management, simulation training delivery, or ICU background. Most of the selected experts had experiences in multiple of these domains. Experts came from different disciplines (Ear Nose and Throat, intensivists, nurses in the intensive department, physiotherapists) and six different hospitals in the French region of Switzerland.

Once the list of experts (n= 16) was finalized, we sent them an invitation by email to explain the study, the objective of the modified Delphi, the course of the different rounds, and the promise of anonymity. An informed consent was provided and completed by the experts before the first round of the modified Delphi. All the experts agreed to participate in the study. Table 1 describes the characteristics of the experts involved in the modified Delphi. The time for completion of each round was four weeks, and email reminders were sent two weeks and three weeks after the first email.

Table 1.

Socio-demographic characteristics of the experts involved in the modified Delphi

|  | | Experts for modified Delphi  N=16 |
| --- | --- | --- |
| Year of practice | |  |
|  | < 5 years | 0 |
|  | 5 – 10 years | 3 |
|  | 11 – 15 years | 5 |
|  | >15 years | 8 |
| Age | |  |
|  | <35 | 1 |
|  | 36-45 | 10 |
|  | 46-55 | 5 |
|  | >55 | 0 |
| Gender |  |  |
|  | Female | 5 |
|  | Male | 11 |
| Simulation background | | |
|  |  | 7 |
| Profession |  |  |
|  | Nurse | 2 |
|  | Physiotherapist | 2 |
|  | Medical doctor | 12 |
| Training background | |  |
|  | Internal medicine | 6 |
|  | Ear Nose and Throat | 2 |
|  | Intensive care | 14 |
|  | Anaesthesiology | 4 |

Round:

During the different rounds, the experts were asked to evaluate the “content relevance” of each item of the assessment scale using a 4-point rating scale from 1 (not important/relevant) to 4 (very important/relevant) [6] (Lynn 1986). The ratings given by the experts were used to calculate the content validity index (CVI) [7, 8]. Expert responses to item-level CVI (I-CVI) scales were binary coded not or somewhat important = 0 and quite or very important =1. An I-CVI score was then calculated for each item. The calculated I-CVI of 0.8 or more indicates the items are relevant, and 0.70 to 0.79 means the item needs revisions. In contrast, less than 0.70 suggests that items need to be eliminated [7].

Experts were also asked to evaluate the “clarity” of each item using a 5-point rating scale from 1 (very poor) to 5 (very good). Expert responses to item-level CVI (I-CVI) scales were binary coded: from level 1 to 3 of the Likert scale = 0 and from level 4 to 5 of the Likert scale =1. The experts' ratings were used to calculate the CVI for each item. The calculated I-CVI of 0.8 or more indicates the items were clear. In contrast, a lack of clarity was demonstrated by an insufficient CVI, below the threshold of 0.8. Items lacking clarity were modified to improve comprehensiveness [9]. Each round required approximately 30 min.

Finally, experts could comment on each item, adding items not present in the actual scale.

The CVIs were used to quantify and determine the content validity of each proposed item.

Finally, a scale level CVI (S-CVI/Ave) score was also calculated. A threshold of 0.8 or higher is considered acceptable. Polit (2006) recommended having an S-CVI/ Ave of .90 or higher.

Results of the modified Delphi rounds:

**Round 1**: The round 1 survey comprised 22 items for Scale number 1, 25 for scale number 2, and 25 for scale number 3.

At the end of the first round, for scale 1 (scenario 1), 20 items had an I-CVI of 0.8 or higher for relevance, and two items had an I-CVI of less than 0.70 for relevance, so these two items were dropped. Experts proposed the addition of 3 items. One item had an I-CVI of 0,56 for clarity, so this item was revised. Finally, 23 items were included in round 2 for the scale 1.

For scale 2 (scenario 2), 23 items had an I-CVI of 0.8 or higher for relevance, and two items had an I-CVI of less than 0.70 for relevance, so these two items were dropped. Experts proposed the addition of 4 items. Finally, 27 items were included in round 2 for the scale 2.

For scale 3 (scenario 3), 21 items had an I-CVI of 0.8 or higher for relevance, two items had an I-CVI between 0.7 and 0.79 for relevance, so these two items were modified, and two items had an I-CVI of less than 0.70 for relevance, so these two items were dropped. Experts did not propose the addition of items. Finally, 23 items were included in round 2 for scale 3.

**Round 2**: At the end of the second round, of the 23 items included for scale 1, all obtained an I-CVI of 0,8 or higher for relevance. Of the 27 items for scale 2, all obtained an I-CVI of 0,8 or higher for relevance. Of the 23 items for scale 3, all obtained an I-CVI of 0,8 or higher for relevance. Scale 1, 2, and 3 received an SCVI/ Ave score of 0.91, 0.9, and 0.94, respectively.

# **Step 3: Design of the final scale and pilot testing**

One member of the educational group previously mentioned (MG), and an intensivist with a background in simulation teaching (GS) tested the three scales by rating five videotaped management for each scenario. Each item was well formulated, and the learners involved in the scenarios clearly observed or explained it aloud. The selected scoring option used two categories: “task performed” or “task not performed” within the recommended timeframe. MG and GS independently rated five participants per scenario using a checklist scale developed for each scenario. The two raters evaluated participants based on their performance in managing scenarios. The intra-class correlation coefficient was computed to assess the agreement between the two raters evaluators in rating the competence levels of 15 participants. There was a strong agreement between the two raters, using the two-way random effect models and “single rater” unit, kappa = 0.975 (95% confidence interval: 0.88-0.99), p < 0.05.

# **Step 4: Final modified Delphi round**

All 16 experts agreed with the three assessment scales.

**Step 5: Item weighting**

As each item is not equally important for the management of the scenario to be successful, experts were asked to rate each item on the three-scale for the success of the management of each scenario from 1 (not important) to 5 (essential). For each item of the three scales, the average of the expert scores was calculated. So, the maximum score for scale 1, 2 and 3 were 101.3, 120.7 and 103, respectively.

**References**

1. Boulkedid R, Abdoul H, Loustau M, Sibony O, Alberti C. Using and reporting the Delphi method for selecting healthcare quality indicators: a systematic review. PLoS One. 2011;6(6):e20476.

2. Flewelling GP, Augustine BD, Groom JA, Kardong-Edgren S, Simmons VC. Design and Validation of a Simulation Scenario and Assessment Tool: A Pilot Project. Aana j. 2023;91(1):46-54.

3. Green R. The Delphi Technique in Educational Research. SAGE Open. 2014;4.

4. Hernandez J, Frallicciardi A, Nadir NA, Gothard MD, Ahmed RA. Development of a Simulation Scenario Evaluation Tool (SSET): modified Delphi study. BMJ Simul Technol Enhanc Learn. 2020;6(6):344-50.

5. Bull C, Crilly J, Latimer S, Gillespie BM. Establishing the content validity of a new emergency department patient-reported experience measure (ED PREM): a Delphi study. BMC Emerg Med. 2022;22(1):65.

6. Lynn MR. Determination and quantification of content validity. Nurs Res. 1986;35(6):382-5.

7. Polit DF, Beck CT. The content validity index: are you sure you know what's being reported? Critique and recommendations. Res Nurs Health. 2006;29(5):489-97.

8. Polit DF, Beck CT, Owen SV. Is the CVI an acceptable indicator of content validity? Appraisal and recommendations. Res Nurs Health. 2007;30(4):459-67.

9. Hennus MP, Nusmeier A, van Heesch GGM, Riedijk MA, Schoenmaker NJ, Soeteman M, et al. Development of entrustable professional activities for paediatric intensive care fellows: A national modified Delphi study. PLoS One. 2021;16(3):e0248565.
